# What Teachers to Avoid

**Published:** 1881-10

**Authors:** 


### WHAT TEACHERS TO AVOID.

The annual recurrence of the Students' number reminds us all of the perpetual rejuvenescence of our art, and of the valiant crowd of workers who press in to fill up the vacancies in our ranks. Veteran after veteran falls, some with a shout of mourning and a hymn of praise making their departure and recording their feats, others lost in oblivion, or surrounded only by the few to whom their labors loved. But the hurrying ranks of young recruits press ever forward; and in the vacancies they see promotion, in the losses opportunity, and in the funeral orations themes for emulation, and the stuff of which ambitions dreams are woven.

This ardour, this courage, these young aspirations and indomitable energies, are the pledge of our future progress. How shall they be directed, and in what quarter shall they make their onslaught with the best chance of victory? We bid them advance whither science beckons: to turn a deaf ear to the easy and plausible blandishments of the "old fogy" school, who fondly dwell on the simple charms of "practical" studies in apprenticeship, and rule of thumb; who talk glibly of the "experience" of years, of the "skilled finger," the "educated touch;" of the "rough and ready" practice of our fathers; of the changes of type; of the good "old-fashioned blue pill and lancet;" who eye ophthalmoscopes askance; who "frankly confess" that they look upon the sphygmograph as a scientific toy: whose urinary cabinet is confined to a bottle of nitric acid, a spirit lamp and a specific gravity bulb; who ask what is the use of the laryngoscope to the general practitioner; to whom the globule-counter and the hæmochronometer are as cabalistic enchantments; who feel doubtful about temperature charts; shirk calculations; and limit their electrical enterprise to turning the wheel of a mechanical "American machine" for paralysed patients. These are the signs by which the bad adviser may be known by the young student; and, once recognized, he should be avoided.—*British Medical Journal*, September 10, 1881.

